# Adrenal Hemorrhage in a Patient Anticoagulated with Apixaban with Antiphospholipid Syndrome

**DOI:** 10.7759/cureus.5108

**Published:** 2019-07-09

**Authors:** Zachary Sanford, Aravinda Nanjundappa, Frank H Annie, Sarah Embrey

**Affiliations:** 1 Cardiology, Charleston Area Medical Center, Charleston, USA; 2 Pharmacy, University of Charleston School of Pharmacy, Charleston, USA

**Keywords:** adrenal hemorrhage, antiphospholipid syndrome, anticoagulation, apixaban, eliquis

## Abstract

Atraumatic adrenal hemorrhage is a rare injury, often due to the disruption of normal hemostasis secondary to sepsis, autoimmune disease, or chronic anticoagulation. We present a case of recurrent adrenal hemorrhage in a patient with antiphospholipid syndrome previously maintained on warfarin for deep vein thrombosis and pulmonary embolism prophylaxis who worsened shortly after transition to apixaban therapy. Initial left-sided adrenal hemorrhage occurred four weeks after beginning apixaban, followed by the development of retinal hemorrhage and later right-sided adrenal hemorrhage. This is, to date, the first reported case of adrenal hemorrhage in a patient receiving chronic anticoagulation with apixaban.

## Introduction

Adrenal hemorrhage presents a diverse yet potentially life-threatening family of injuries of variable etiology, including trauma, life-threatening bacterial sepsis, antiphospholipid antibody syndrome (APS), heparin-induced thrombocytopenia (HIT), chronic anticoagulant use, disseminated intravascular coagulation (DIC), and multiorgan failure [1-4]. Among these, bleeding diatheses concurrent bilateral adrenal hemorrhage presents a potentially catastrophic injury most frequently ascribed to disseminated bacterial infections involving *Neisseria, Pneumococcus*, and *Staphylococcus* spp. that, if not recognized and treated promptly, have a high rate of mortality [5-8]. However, throughout the literature, there are rare but reported cases of non-infectious etiologies for bilateral adrenal hemorrhage, including in the postoperative setting or as a function of abnormalities of normal hemostasis [9-10].

With regards to aberrations in functional hemostasis, the precise mechanism by which APS leads to adrenal hemorrhage is not yet fully elucidated. The current literature suggests antiphospholipid antibodies adhere to phospholipid-binding plasma proteins, promoting thrombosis via dysregulation of the balance between coagulation and fibrinolysis [11-12]. The resulting adrenal vein thrombosis is speculated to be secondary to immune complex accumulation, disruption of adrenal gland outflow, and ultimately hemorrhage into the adrenal gland proper [11-15]. Such a precarious environment can be further complicated by chronic anticoagulation, which interrupts normal hemostasis such that hemorrhages can often prove disastrous [16].

The evolving landscape of pharmacologic anticoagulation is continuing to produce newer agents whose effectiveness and safety profiles are only beginning to be fully understood. One such novel anticoagulating agent is apixaban (trade name Eliquis®), a direct factor Xa inhibitor approved by the Food and Drug Administration (FDA) in 2014 for the treatment of acute deep vein thromboses (DVT) and pulmonary embolisms (PE) in addition to risk the reduction of subsequent events, and apixaban is also approved for anticoagulation in nonvalvular atrial fibrillation [17-19]. These claims have been supported by the AMPLIFY (Apixaban for the Initial Management of Pulmonary Embolism and Deep-Vein Thrombosis as First-Line Therapy) trial in addition to broader claims of reductions in hospitalization times as well as overall improved outcome measures when compared to warfarin. This agent serves as an attractive alternative to warfarin because although it is dosed twice daily, it has a smaller side-effect profile and requires no routine monitoring of coagulation profiles [18]. Although effective as a newly introduced agent in the clinical management of DVT and PE, potential complications remain. The following case presents a potential cause for such consideration, where an individual with APS stably managed with warfarin was transitioned to apixaban, subsequently incurring bilateral adrenal hemorrhage.

## Case presentation

Consent to discuss the following was obtained. A 42-year-old Caucasian male presented to our hospital complaining of acute onset severe right flank pain first manifesting while repositioning himself in bed earlier that same day. Subjective assessment of the pain measured 8/10 intensity with no radiation or associated symptoms of gastrointestinal or genitourinary involvement, although the patient was previously febrile to 39.1°C one day prior to admission. Past medical history was significant APS, DVT, and PE controlled with chronic anticoagulation on warfarin for approximately 10 years until the patient’s primary care physician changed his medication to apixaban. One month later, the patient suffered left-sided adrenal hemorrhage, at which point apixaban was discontinued and warfarin was resumed. Nine months later, the patient experienced right-sided flank pain and reported this pain to mimic symptoms present during a previous episode of adrenal hemorrhage.

**Figure 1:**
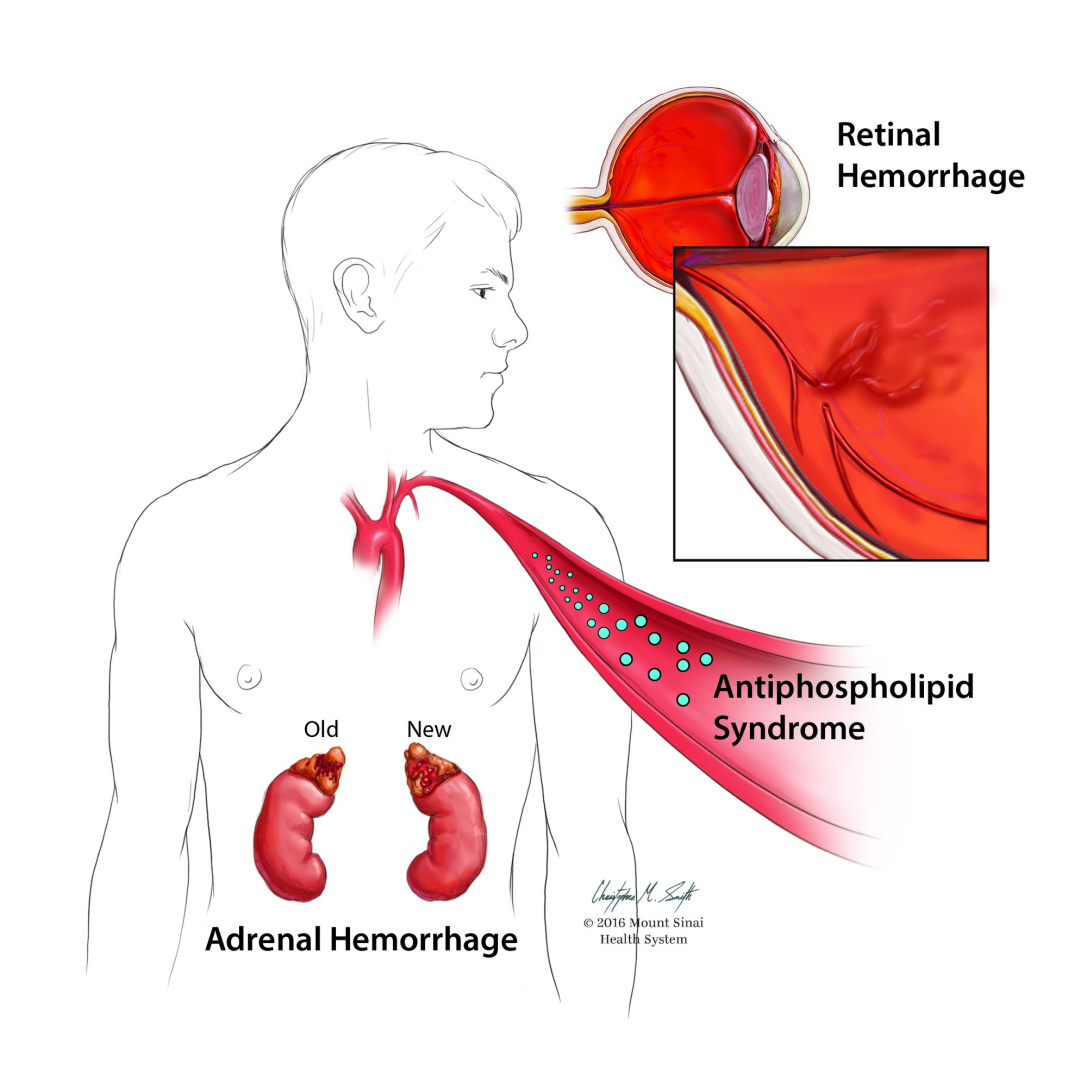
Adrenal hemorrhage This image was requested and completed by Mount Sinai Beth Israel, 2016.

Computed tomography (CT) imaging of the abdomen and pelvis revealed 4.5 x 3.0 cm right-sided adrenal hemorrhage. At the time of the evaluation, the patient had already been evaluated by his ophthalmologist for a new-onset blurring of vision in the right eye, determined to be the result of retinal hemorrhage as described in Figure 1. Concerns for possible Waterhouse-Friderichsen syndrome were based on a concurrent left plantar foot ulcer, which was culture positive for methicillin-sensitive *Staphylococcus aureus, Enterococcus faecalis,* and *Pseudomonas aeruginosa *being managed with vancomycin and ciprofloxacin. These concerns were eventually ruled out with negative blood cultures. The patient was found to be thrombocytopenic likely due to APS, anemic due to chronic disease, and had previously been tested positive for lupus anticoagulant, antinuclear antibody, and anticardiolipin IgA. The patient was determined to have subtherapeutic anticoagulation with an international normalized ratio (INR) of 1.32 at admission, reporting that on weekly evaluations, he was below the target INR of 2.0 for approximately four weeks. Heparin bridged to warfarin was resumed with a new target INR of 2.5 to 3.5 to reduce the possibility of recurrent thromboembolic events.

## Discussion

This case presents the first reported event of adrenal hemorrhage in a patient undergoing anticoagulation with apixaban. Inherent in the use of new anticoagulants, such as apixaban, for chronic anticoagulation is the lack of sufficient data regarding efficacy in the setting of APS. Existing literature is scant on establishing an ideal dosing regimen for APS or other subgroups of hypercoagulable states, which may result in higher rates of adverse effects, such as bleeding, in this population. In May 2018, the FDA approved the first factor Xa antidote product in the United States, but due to high costs, this may not be widely available in institutions. Work by Arachchillage et al. highlights that although challenges remain in the use of warfarin in patients with APS, utilizing agents such as apixaban remains favorable for these patients due to insufficient clinical data [18]. The chronic management of APS patients presents unique challenges that may not directly coincide with the mechanisms by which apixaban affords anticoagulation benefits. This does not, however, rule out the potential for benefit in the chronic management of APS patients, although it warrants further study and the development of specific protocols as to the implementation of apixaban.

Measuring the risk of further thrombosis and loss of function without anticoagulation must be weighed against risks of bleeding and should be discussed with patients on a case-by-case basis. Patients experiencing adrenal hemorrhage who are not in acute distress are often associated with elevated coagulation risk, which can be further exacerbated by the presence of lupus anticoagulant or pharmacologic anticoagulation [13]. Therefore, the resumption of anticoagulation in APS patients should begin as soon as possible upon resolution of acute adrenal hemorrhage. However, of specific concern to the patient presented in this case, there are currently no definitive guidelines in the literature regarding the ideal duration to postpone anticoagulation status post-retinal hemorrhage in chronically anticoagulated patients. For this patient, we utilized the Naranjo nomogram, a validated scoring system used to determine the likelihood of whether an adverse drug reaction is due to the drug itself rather than as a result of other concomitant factors [20]. The resulting score was four points, which indicates that the adverse reaction of bleeding is considered to have a possible correlation with the drug in question, which was apixaban in this case. The nomogram, along with the resulting score from this patient case is included in Table 1. We feel that this result further reinforces the likelihood that apixaban contributed to this patient’s adrenal hemorrhage, and that providers should be aware of this potential adverse effect in patients receiving the medication.

**Table 1:** Naranjo Nomogram with Case Report Score

	Scores			
Scoring Parameters	Yes	No	Don’t Know	Case Report Score
Are there previous conclusive reports of this reaction?	+1	0	0	0 (No)
Did the adverse event occur after the suspected drug was given?	+2	-1	0	+2 (Yes)
Did the adverse event improve when the drug was discontinued or a specific antagonist was administered?	+1	0	0	0 (No)
Did the adverse reaction reappear when the drug was given again?	+2	-1	0	0 (Don’t Know)
Are there alternative causes that, on their own, could have caused the reaction?	-1	+2	0	+2 (No)
Did the reaction appear after placebo was given?	-1	+1	0	0 (Don’t Know)
Was the blood level detected known to be toxic?	+1	0	0	0 (Don’t Know)
Was the reaction more severe when the dose was increased or was the reaction less severe when the dose was decreased?	+1	0	0	0 (Don’t Know)
Did the patient have a similar reaction to the same or similar drugs in the past?	+1	0	0	0 (Don’t Know)
Was the adverse event confirmed by any objective evidence?	+1	0	0	0 (No)

## Conclusions

In summary, we present a case of recurrent adrenal hemorrhage accompanied by retinal hemorrhage precipitated during a transition from warfarin to apixaban. Due to the relative scarcity of data in assessing efficacious dosing for chronic anticoagulation with apixaban and the course of thromboembolic events this patient underwent as a result of interrupting a 10-year course of successful therapy on warfarin, we suggest caution in altering pre-established treatment modalities. As apixaban is a new agent in the management of acute and chronic DVT and PE with little to no literature specifically studying its effects in patients with APS, we recommend continuing to use warfarin as the standard of care in chronic anticoagulation in APS.
